# Greenhouse Gas Emissions in Dairy Goat Farming Systems: Abatement Potential and Cost

**DOI:** 10.3390/ani9110945

**Published:** 2019-11-10

**Authors:** Alexandra Sintori, Irene Tzouramani, Angelos Liontakis

**Affiliations:** Agricultural Economics Research Institute (AGRERI), Hellenic Agricultural Organization (DEMETER), PC. 11528 Athens, Greece; al_sintori@agreri.gr (A.S.); tzouramani@agreri.gr (I.T.)

**Keywords:** dairy goat farming, linear programming, GHG emissions, abatement cost, mitigation options, carbon footprint

## Abstract

**Simple Summary:**

Agriculture and particularly livestock farming is associated with the production of certain gases that contribute to global warming, commonly referred to as greenhouse gases. These gases are the result of the use of machinery and other inputs such as fertilizers and pesticides or are associated with the digestion process of animals. In this work, we have analyzed data from dairy goat farms in Greece to estimate the amount of greenhouse gases per kilogram of milk produced and identify farming practices that can result in their reduction. We found that greenhouse gases per kilogram of milk are fewer in farms that are characterized by higher milk production per goat. Furthermore, certain practices like the use of homegrown feed instead of purchased feed and the use of compound feedstuffs or oil-rich feedstuffs like cottonseed cake can result in lower greenhouse gases in goat farms. Also, the analysis suggests that the reduction of greenhouse gases can lead to a reduction of farm income, especially in the case of intensive farms. This finding has to be taken into consideration by policy makers and possible measures to compensate for this income loss have to be explored.

**Abstract:**

Dairy goat farming is an important agricultural activity in the Mediterranean region. In Greece the activity offers occupation and income to thousands of families mainly located in mountainous and semi-mountainous areas of the country where it utilizes low productivity pastures and shrub lands. Furthermore, goats are more resilient to climate changes compared to other species, and are often characterized as ideal for keeping in drought areas. However, there is still limited evidence on total greenhouse gases (GHG) emitted from goat farms and their mitigation potential. In this context, this study aims to estimate GHG emissions of goat farms in Greece and explore their abatement options using an economic optimization model. Three case studies are explored i.e., an extensive, a semi-intensive and an intensive goat farm that correspond to the main goat production systems identified in Greece. The analysis aims to assess total GHGs as well as the impact of abatement on the structures, gross margins and labor inputs of the farms under investigation. The issue of the marginal abatement cost is also addressed. The results indicate that the extensive farm causes higher emissions/kg of milk produced (4.08 kg CO_2_-eq) compared to the semi-intensive and intensive farms (2.04 kg and 1.82 kg of CO_2_-equivelants, respectively). The results also emphasize the higher marginal abatement cost of the intensive farm. In all farm types, abatement is achieved primarily through the reduction of the livestock capital and secondarily by other appropriate farming practices, like substitution of purchased feed with homegrown feed.

## 1. Introduction

Goat farming is an important agricultural activity in Greece since it is mainly located in less favored areas of the country where it utilizes low-productivity pastureland and shrubland. It is estimated that goat farming yields income for 64,049 Greek farms that breed over 3.5 million goats [1]. The activity aims primarily at the production of milk and secondarily at the production of meat. According to Kitsopanidis [2], milk is on average responsible for over 70% of gross revenue of dairy goat farms, with the exception of very hardy, local breeds. It is estimated that 75% of the Greek goat milk is used for the production of cheeses, especially Feta. Furthermore, the activity contributes highly to regional development and helps maintain the population in depressed and marginal areas. Therefore, the preservation of the activity and the income it yields is important not only for farmers but also for policy makers.

The prevailing goat farming system in the country is the extensive one with or without transhumance, in which the nutrition of the livestock is based on grazing. The main characteristic of the extensive breeding farms is the low invested capital and the low-productivity livestock, consisting mainly of native races [3]. More modern and intensive farms that aim to increase their productivity through supplementary feeding, mainly from on-produced cereals and forage, are also present. Specifically, three commercial goat production systems are identified in Greece, namely the traditional extensive farming system, the semi-intensive farming system and the intensive farming system [2]. As mentioned above, in the extensive farming system feed requirements are met mainly through pasturing. In the semi-intensive farming system additional supplementary feed is provided, while in the intensive farming system no pasture is utilized. This heterogeneity among the alternative goat farming systems results in differences in their socioeconomic as well as their environmental sustainability.

One of the main environmental issues associated with livestock farming is the emission of greenhouse gases (GHGs). GHG emissions are particularly high in the case of ruminant livestock farming because of methane production through enteric fermentation [4,5]. The issue of GHG emissions in livestock farms has been addressed in a number of studies that focus mainly on cattle farms [6,7,8,9]. On the other hand, studies that focus on the emission of GHGs from sheep and goat farms refer mainly to meat and wool production farming systems that have different technical and economic characteristics from dairy farms (e.g., [10,11]). In the case of small ruminant dairy farming in Greece, limited studies on GHG emissions from sheep farms and their abatement potential are available (see [12]).

This study aims to address the issue of GHG emissions in dairy goat farms, using an economic optimization model, developed to capture and represent the structure and function of Greek goat farms. The use of such model in GHG studies has the advantage that it accounts for all possible sources of GHG emissions in goat farms and therefore reduced emissions from one source at the optimal solution does not result in increased emissions from other sources. Furthermore, since the model is an optimization model, abatement options are explored within the context of gross margin maximization. In other words, abatement practices and options that are proposed by the model are the least-cost options for the farms. Furthermore, this cost is precisely estimated and marginal abatement cost curves are derived. Thus, the analysis and the results it yields can be useful not only for agriculturalists but also for farmers, agricultural advisors and policy makers.

## 2. Materials and Methods

Optimization models, and specifically linear programming (LP) models are commonly used in agricultural studies (e.g., [13,14,15,16]). They yield the optimal amongst all feasible farm plans, taking into account technical and agronomic constraints of the farms. When the matter of GHG emissions in livestock and crop livestock farms is addressed, the complexity of the farm operation, the multiple sources of emissions, and the substitution possibilities between alternative activities require the use of a model that can capture all the interrelationships of these activities. That said, a number of studies use LP models to assess GHGs from various sources and identify cost-effective mitigation strategies (e.g., [9,10,17,18,19,20,21]).

The general expression of a linear programming model is as follows [22]:

(1) $$\mathrm{Max}\;g\left(x\right)=z=c_{1}x_{1}+c_{2}x_{2}+\ldots +c_{n}x_{n}$$

Subject to the constraints:

$$a_{11}x_{1}+a_{12}x_{2}+\ldots +a_{1n}x_{n}\leq b_{1}$$

$$a_{21}x_{1}+a_{22}x_{2}+\ldots +a_{2n}x_{n}\leq b_{2}$$

$$a_{m1}x_{1}+a_{m2}x_{2}+\ldots +a_{mn}x_{n}\leq b_{m}$$

$$x_{j}\geq 0$$

where $x_{j}$ (j= 1, 2,…, n) are the decision variables of the model, they are unknown and determined by the model according to what maximizes gross margin (e.g., number of productive goats, hectares of cereals or forages etc.), $c_{j}$ are known economic parameters (e.g., gross margin per unit of activity $x_{j}$), $a_{ij}$ (i= 1, 2,…, m) are known technical parameters (e.g., hours of labor or variable inputs per activity $x_{j}$) and $b_{i}$ are also known parameters that express the availability of inputs (e.g., maximum available labor, capital or land inputs).

The characteristics of the optimization model that was used in this analysis is described in more detail in the following paragraphs. The data used in the analysis is also presented in the same section.

### 2.1. Model Specification

**Figure 1:**
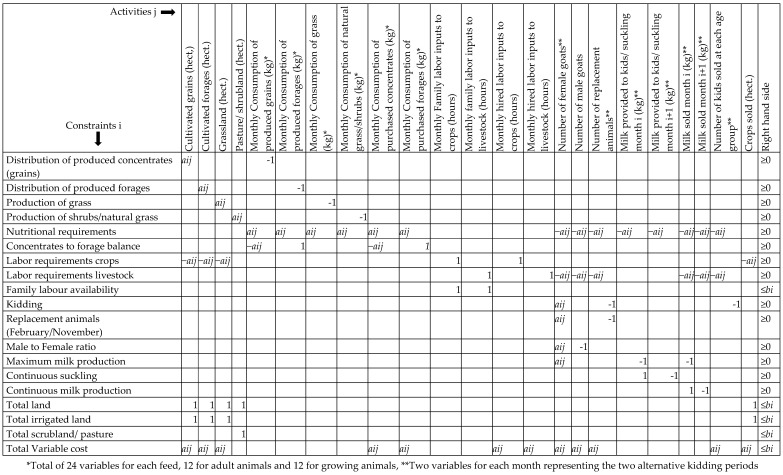
Representation of the original linear programming model.

The model used in this analysis has 241 decision variables and 236 technical and economic constraints and it is presented in Figure 1 which represents the LP matrix. The decision variables of the model (Activities *i* in Figure 1) can be grouped in three main categories. The first one includes all the decision variables that refer to crops, pasture, grassland, shrubland and feeding i.e., to the distribution of produced and purchased feed. The second category refers to labor variables, and the final category to livestock and product variables. In addition to the non-negativity constraint of all decision variables, variables which refer to the livestock capital are restricted to receive only integer numbers and therefore the model is in fact a mixed-integer programming model. The constraints of the model (Constraints *j* in Figure 1) refer mainly to the feeding of the livestock but also to the availability of labor, land and other inputs. In Figure 1, the constraints of the model and their technical parameters (*α_ij_*) as well as their right hand side parameters (*b_i_*) are presented in all lines except for the first line that presents the activities (*x_j_*).

The model is built to accurately reflect the structure and function of Greek goat farms and allocates all their available inputs and resources to the alternative economic activities and practices according to what maximizes their total gross margin (objective function). Therefore, the farm plan it suggests is in economic terms the optimal. It should be noted that the model is built according to the dairy sheep model described in detail in Sintori [23].

#### 2.1.1. Crop, Pasture, Grassland and Feeding Variables

Crop activities of the goat farms involve forage and grain production for livestock feeding but also crop production for sale. The main crops cultivated in Greek goat farms are maize for grain and alfalfa for hay. Other crops may also be cultivated like maize for silage or barley for grain. For each crop, pasture and grassland used, one variable is included in the model that expresses the land in hectares allocated to the activity. The economic parameter of the specific variable is negative and expresses the cultivation cost per hectare excluding labor which is represented by a different set of variables. Feeding variables are defined according to month, type of feed and livestock category. In other words, the consumption of each feed (e.g., produced maize, purchased maize, produced alfalfa hay, purchased mixtures) is presented monthly and for two separate categories of livestock i.e., growing and adult animals. This structure allows the model to simulate farm decision making regarding the distribution of homegrown feed and purchase of additional feeds throughout the year. Furthermore, the use of such detailed data on feeding practices of the farms allows the model to be accurate and realistic regarding the predicted type and amount of feed required by the livestock, but also the methane emitted from enteric fermentation. Finally, it should be emphasized that the economic parameters of the variables that refer to purchased feed are negative and represent the price per kilo for each feed, while the economic parameter of each variable that refers to the consumption of homegrown feed is zero, since the cost of production is in fact the economic parameter of the related crop variable and, therefore, it has already been accounted for.

#### 2.1.2. Labor Variables

In order for goat farms to operate one main required input is labor. In Greece, goat farms are in their majority traditional and family owned with very low mechanization degree, since extensive and semi-intensive farms rarely even use milking machines. The amount of labor inputs required in these farm types are also increased because of grazing. Labor variables incorporated in the model represent the amount of labor inputs required each month of the year in hours. The model allocates the available family labor between all crop and livestock activities of the farm. Variables representing the additional hired labor required each month in the above activities are also incorporated in the model. The economic parameters of these variables are negative and represent the wage per hour in livestock and crop activities.

#### 2.1.3. Livestock and Product Variables

Livestock variables incorporated in the model refer to the number of female and male goats, replacement animals and kids that constitute the livestock of the farm. Two variables are used to represent the female goats that are kept in the livestock representing two different kidding periods. One variable refers to the goats that give birth in late November and the other to the goats that give birth in late February. These two kidding periods were chosen to reflect the practices of Greek goat farms, that aim to satisfy the increased demand for goat meat during Christmas and Easter. The economic parameters of these variables are negative and represent the annual variable cost of keeping and breeding one animal for one year, except for labor and feeding cost, since both labor and feeding are represented by the variables already described in previous paragraphs. As far as kids are concerned, they are represented in the model by a set of variables according to their age group in months. Specifically, 12 variables are used in the model to reflect the young kids between the ages of one and six months born during the alternative two kidding periods. The economic parameters of these variables are positive and express the gross margin per animal sold at each specific age. As mentioned above, all livestock variables are allowed to receive only integer numbers.

The final set of variables incorporated in the model represents milk production per month and per kidding period. Milk variables include variables that refer to suckling as well as variables that refer to milk production for sale. The economic parameter of the milk for sale variables is positive and expresses milk price.

#### 2.1.4. Feeding Constraints

The main component of the model reflects the balance of the monthly feed requirements of the livestock. Minimum intake of dry matter, net energy of lactation, nitrogen and fiber matter is ensured through monthly constraints. The feed requirements of the livestock are estimated according to Zervas et al. [24] (see Table 1). For the female productive goats these feed requirements include requirements for preservation, activity and pregnancy. Extra requirements for lactation are estimated per kilogram of produced milk. For male productive goats, the requirements refer to their preservation, activity and reproduction. For the replacement animals, the feed requirements are estimated every month taking into account the live-weight increase. The weight increase is also taken into account in the case of the kids, for which feed requirements are estimated for the period that they remain in the farm.

On-produced feed crops, external feed inputs, available grassland and pastureland/shrubland are used for the balance of the feed requirements of the flock. The composition and the nutritional value per kilogram of feedstuff is taken from Kalaisakis [25], Jarrige [26], Zervas et al. [24] and Feedipedia [27] (Table 2). The nutritional value and the production of grassland and pasture are estimated taking into account Papachristou [28], Zervas et al. [24], F.R.I. [29], Platis and Papanastasis [30], Platis et al. [31]. Additional monthly constraints are incorporated in the model to ensure minimum and realistic intake of concentrate feeds, according to the feeding practices of the farms.

#### 2.1.5. Additional Constraints

Another component of the model ensures that monthly labor requirements of all production activities are balanced, mainly with family labor inputs. Additional hired labor can be used, if necessary, in both livestock and crop activities.

Land constraints are also incorporated in the model to ensure that the total area utilized by the various crop activities, grassland and pastureland/shrubland is smaller than the available land of the farm. Moreover, one land constraint refers to total available irrigated land of each farm and another to total available pastureland/shrubland. A final set of constraints reflects the demography of the livestock and the maximum milk and meat production capabilities per goat.

#### 2.1.6. Greenhouse Gas (GHG) Emissions

**Figure 2:**
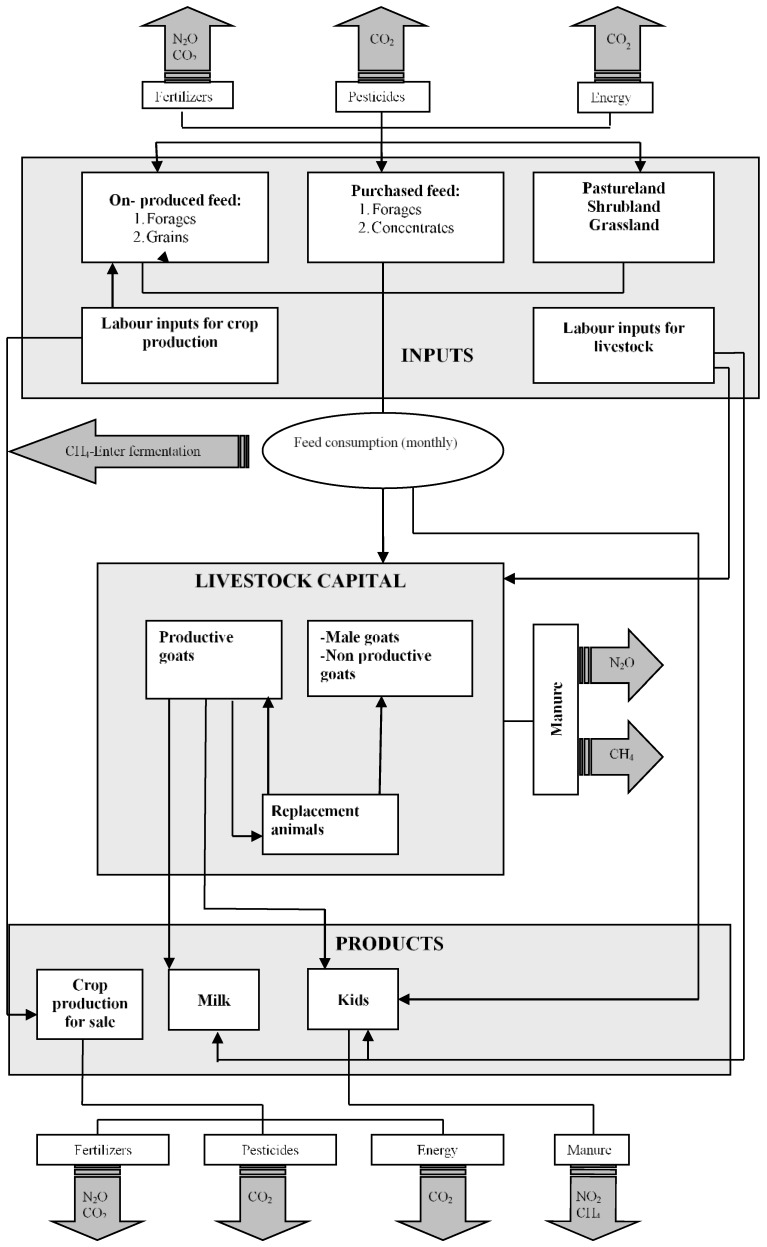
Graphical representation of the mathematical programming model and the emission sources considered in the analysis.

In order to accurately derive mitigation options for the goat farms, it is important to identify all potential sources of GHGs related to the activity, and include them in the model. The main GHGs, in livestock farms are methane (CH_4_) from enteric fermentation and methane and nitrous oxide (N_2_O) from manure. In addition, in a crop-livestock farm, nitrous oxide emissions (N_2_O) from nitrogen fertilizers should also be accounted for (see for example [10,32]). Carbon dioxide emissions (CO_2_) from the use of machinery are an additional source of GHGs. A graphical representation of the model used in the analysis and the emission sources it includes is presented in Figure 2. The emissions sources that the model takes into account are also summarized in Table 3.

It should be noted that CH_4_ and N_2_O have been converted to CO_2_-equivalents (CO_2_-eq) using the conversion factors proposed by the Intergovernmental Panel on Climate Change (IPCC) [4] i.e., 1 kg of N_2_O = 298 kg of CO_2_-eq and 1 kg of CH_4_ = 25 kg of CO_2_-eq. The method used to estimate emissions from various sources in the goat farms is described in more detail in the following paragraphs. Emissions from all sources estimated as CO_2_-equivalents are added together to estimate total GHG emissions of the goat farms.

Methane production from enteric fermentation is the most important source of GHGs in small ruminant livestock farms and it is associated with the feeding practices of each farm. Farmers choose to feed their livestock with on-produced feed and purchased feed taking into account their cost and their nutritional value. Linear programming models select the optimal combination of feedstuff and suggest the ration that helps maximize gross margin (least cost ration). For this reason, the ration used in this analysis is not fixed and methane emissions are predicted from intake, taking into account the requirements of the livestock estimated as previously described and the composition of feedstuff, that can be found in Table 2 (see also [33,34]). Specifically, for each of the variables that refer to feed consumption the methane emissions per kilogram have been estimated and included in the model as the technical parameter of this variable in a new constraint regarding GHG emissions. To estimate the percent of gross energy intake lost as methane from enteric fermentation (*ECH_4_/EB*) the following equation was used [33]:

(2) $$ECH_{4}/EB=9.84-0.0461ADL-0.0509EE+0.00366St+0.00648CP$$

where: *ADL* = g of lignin/kg of DM, *EE* = g of ether extract/kg of DM, *St* = g of starch/kg of DM and *CP* = g of protein/kg of DM.

Methane emissions from manure are estimated using the Tier 2 methodology proposed by the IPCC [4], which takes into account the management system of manure and the energy consumption of livestock (Equation (3)).

(3) $$EF=\left(VS\cdot 365\right)\cdot \left[B_{o}\cdot 0.67kg/m^{3}\cdot \underset{S,k}{\sum }\frac{MCF_{S,k}}{100}\cdot MS_{\left(S,k\right)}\right]$$

where: *EF* = annual methane emissions from manure (kg CH_4_/head/year), *VS* = daily volatile solid excreted (kg of dry matter/head/day), *B_o_* = maximum methane producing capacity for manure produced (0.18 m^3^ CH_4_/kg VS for Greece), *MCF_(S,k)_* = methane conversion factors for each manure management system and climate region (1.5% for Greece), *MS_(S,k)_* = fraction of manure handled using manure management system S to climate region k (estimated for each farm according to their farming practices) and 365 are the days within the year.

VS is estimated from the gross energy intake (GE) expressed in MJ/head/day, the digestibility of the feed (DE/100) e.g., 65%,the ash content of manure (ASH/100) (8% for goats according to the IPCC) and the conversion factor for dietary GE per kg of dry matter (18.45 MJ/kg), using Equation (4):

(4) VS=GE/18.45⋅(1−DE/100)⋅(1−ASH/100)

The methodology to estimate the energy requirements per livestock category, which is necessary for the implementation of the Tier 2 methodology has already been presented.

Direct N_2_O emissions from manure management and pastureland are estimated according to the Tier 1 methodology [4], using the live weight of each livestock category (Equation (5)):

(5) $$N_{2}O_{D\left(mm\right)}=\frac{44}{28}\cdot \underset{S}{\sum }Nex_{}\cdot MS_{\left(S\right)}\cdot EF_{\left(S\right)}$$

where: *N_2_O_D(mm)_* = direct Ν_2_Ο emissions from manure management kg/year/head, *N_ex_* = annual N excretion (kg of N/head/year), *EF_(s)_ =* emission factor for direct Ν_2_Ο emissions from manure management system *S* (kg N_2_O-N/kg N). *EF_(s)_* equals 0.02 kg N_2_O-N/kg N when manure is managed in solid storage and 0.01 kg N_2_O-N/kg N when manure is deposited on pasture [4]. It should be noted that according to the IPCC guidelines Ν_2_Ο emissions generated by manure deposited on pastures is reported under *Emissions from managed soils*. In this analysis, however, these emissions have been considered, so that comparison between grazing and housed animals can be made.

*N_ex_* is estimated taking into account the typical animal mass (TAM) in kg/head and the N excretion rate using the equation (*N_rate_* for goats = 1.28 kg of N/1000 kg of animal mass/day):

(6) $$N\mathrm{ex}=N_{rate}\cdot \frac{TAM}{1000}\cdot 365$$

where 365 are the number of days within the year.

According to the IPCC (2006), for the estimation of indirect Ν_2_Ο emissions, first the fraction of Ν that volatilizes as ΝΗ_3_ and ΝΟ_x_ is estimated according to Equation (7) and then the amount of manure nitrogen that is lost due to volatilization of NH_3_ and NOx is estimated using Equation (8):

(7) $$N_{volatilization-MMS}=\underset{S}{\sum }Nex\cdot MS_{\left(S\right)}\cdot Frac_{GasMS,\left(s\right)}$$

(8) $$N_{2}O_{G\left(mm\right)}=\left(N_{volatilization-MMS}\cdot EF_{4}\right)\cdot \frac{44}{28}$$

where: MMS stands for manure management system, *Frac_GasMS(s)_* = is the Fraction Ν that volatilizes as ΝΗ_3_ and ΝΟ_x_ (0.12) and *EF*_4_ = emissions factor for Ν_2_Ο from Ν that volatilizes (0.010 N_2_O-N/kg ΝΗ_3_-N + ΝΟ_x_ –N volatilized).

In our analysis, we have also included direct and indirect N_2_O emissions from the use of nitrogen fertilizers. First, the total amount of nitrogen applied in fields has been calculated using the amount and the type of fertilizer (see also [17,34]). Then direct and indirect emissions from the applied N have been estimated according to the Tier 1 methodology and the emission factors proposed by the IPCC [4].

Carbon dioxide linked to energy use is another GHG of crop-livestock farms. The main sources of energy in these farms are fuel (mainly diesel) and electricity (see also [7]). To estimate the emissions from energy use, fuel or electricity requirements for every farm operation and type of machinery are accessed and multiplied by appropriate emission factors [10]. Specifically, as far as electricity is concerned and due to the fact that in Greece lignite is used for the production of electricity, the emission factor considered in the analysis is quite high (0.855 kg of CO_2_-eq per KWh). The emission factors per liter of petrol and diesel used in the analysis are 2.23 and 2.66 kg of CO_2_-eq, respectively.

Other inputs, like fertilizers and pesticides have also caused GHG emissions when they were manufactured. These emissions have been taken into account as well, using farm-level data to estimate the amount of inputs used and related literature to estimate the emissions caused by the manufacture of these inputs. Carbon dioxide emissions from the manufacture of fertilizers are assumed 0.3 kg of CO_2_ eq/kg of N, 0.9 kg of CO_2_ eq/kg of P and 0.6 kg of CO_2_ eq/kg of K. The energy requirements for the manufacture of herbicides, insecticides and fungicides are 287 MJ/kg, 263 MJ/kg and 195 MJ/kg, respectively [35,36,37,38,39]. Emissions are then calculated by multiplying the total energy requirements with 0.069, which is the amount of CO_2_ produced per MJ of energy consumed.

Other pre-chain emissions have also been estimated and included in the analysis, following the work of Olesen et al. [7]. As mentioned above, farmers choose whether to feed their livestock with on-produced or purchased feed. Therefore, N_2_O emissions from nitrogen fertilizers and CO_2_ emissions from energy requirements have also been estimated per kilogram of purchased feed, according to the methodology that has already been presented in the previous paragraph. However, to estimate the amount of inputs (e.g., fertilizers) required for the production of the purchased feed data from 150 farms producing these feeds and operating in Continental Greece have been used. The data is part of a larger data set obtained during the implementation of the program “Search for Innovative Occupations of Tobacco Producers in the Rural Sector (Measure 9, Reg (EU) 2182/02)” and involve detailed information regarding the practices used to produce feedstuff commonly purchased by goat farms.

The original optimization model presented in Figure 1 was used to obtain the optimal farm plan of the goat farms. GHG emissions from various sources and total GHG emissions were then estimated at this optimal solution and used as the basis of our estimations (0% abatement level). The second step of our methodology is to derive the optimal farm plan across increasing levels of abatement, and assess impact on farm structure and gross margin. Following a number of studies (e.g., [17,23,40]), this was achieved by inserting an additional constraint in the model. Specifically, if a is the level of abatement (a < 1) and *e*_0_^*^ the total emissions at the optimal farm plan, then a new constraint is inserted in the model which restricts total farm emissions below (1 − a)*e*_0_^*^. The shadow price of this constraint is used to estimate GHG marginal abatement cost for each production system. Additionally, marginal abatement cost curves are derived for each farm type. In order to obtain the marginal abatement cost curves the right-hand side parameter of the emissions constraint was reduced marginally i.e., 1 tone, and the impact on gross margin was estimated. This procedure was performed a number of times to derive the cost curve. It should be emphasized that this kind of sensitivity analysis is usually performed automatically in LP models, but in this analysis some variables are restricted to receive only integer numbers. Therefore, sensitivity analysis could not be performed automatically and marginal costs were obtained manually.

### 2.2. Materials

To estimate the parameters of the model (*cj*, *aij* and *bi*) data from actual goat farms were used. Studies that implement the LP methodology commonly utilize data from representative or typical farms of the region under study (see for example [14]). LP models are not statistical models and, therefore, data from a large number of farms is not usually required. On the other hand, to increase the predictive ability of such models detailed data was used and the model was validated through the comparison of the predicted values (optimal solution) and the actual values of the representative farms. In this analysis, data from three goat farms were used. The goat farms were selected to represent the common production systems identified in Greece. Table 4, summarizes the main characteristics of these production systems as described in the literature. All of the above characteristics were taken under consideration during the selection of the goat farms, the characteristics of which are also presented in Table 4. All farms are located in Continental Greece, specifically the intensive and the extensive farms, were located in the region of Thessaly (Prefectures of Karditsa and Magnesia, respectively) and the semi-intensive farm in the region of Epirus (Prefecture of Preveza).

As can be seen in Table 4, the livestock of the extensive farm consists of hardy local breeds that are characterized by low productivity. More specifically, the farm breeds 350 reproductive female goats with an annual production of milk of 115 kg/goat. The average live-weight of the goat is 50 kg and the prolificacy index is 1.2.

The farm produces barley and uses only 2 hectares of grassland. Feeding of the livestock is based mainly in pasturing, since the farm uses 100 hectares of summer pasture and 50 hectares of winter pasture (mainly shrub cover). Additional feed is purchased, mainly maize, cotton seed and alfalfa. Additional parameters used in the model regarding the extensive farm can be found in Table 5.

The livestock of the semi-intensive farm consists of 300 female productive goats, with an annual milk production of 300 kg/goat and a prolificacy index of 1.5. The farm maintains maize cultivation for grain production and utilizes 50 hectares of pastureland (mainly shrub cover). Additional purchased feed is used, namely maize, barley, alfalfa and ready to buy feed mixes for goats. Table 5, summarizes the main technicoeconomic characteristics of the semi-intensive farm.

Finally, the intensive farm has a livestock of 300 female productive goats with an annual production of milk of about 520 kg/goat, an average live-weight of 70 kg and a prolificacy index of 1.8. For the feeding of the highly productive livestock, maize for grain and forage production, alfalfa and barley are cultivated. Additionally, special feed mixtures and alfalfa is purchased (see also Table 5).

The detailed technical and economic data required from the three farms were obtained in the summer of 2015 and refer to the year 2014.

## 3. Results

**Figure 3:**
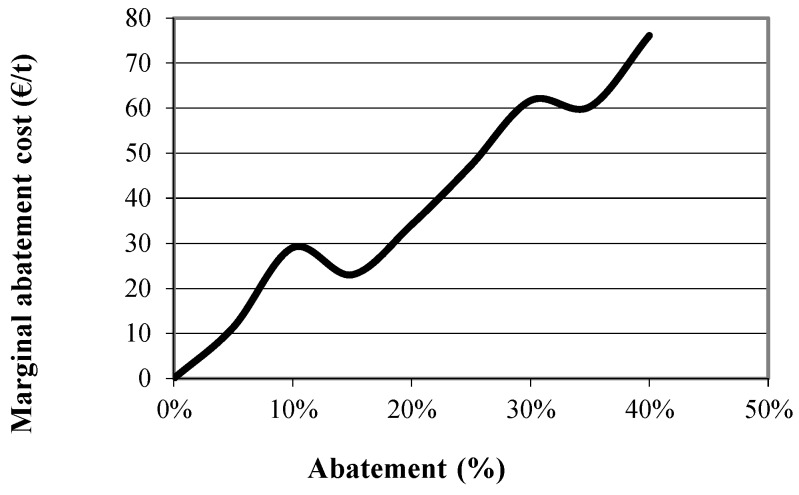
Marginal abatement cost curve of the extensive farm.

**Figure 4:**
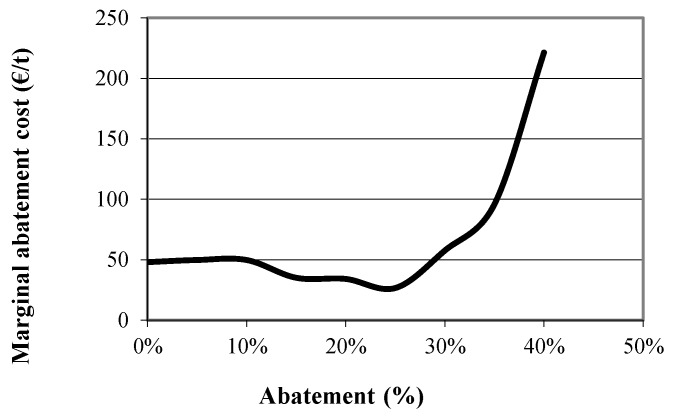
Marginal abatement cost curve of the semi-intensive farm.

**Figure 5:**
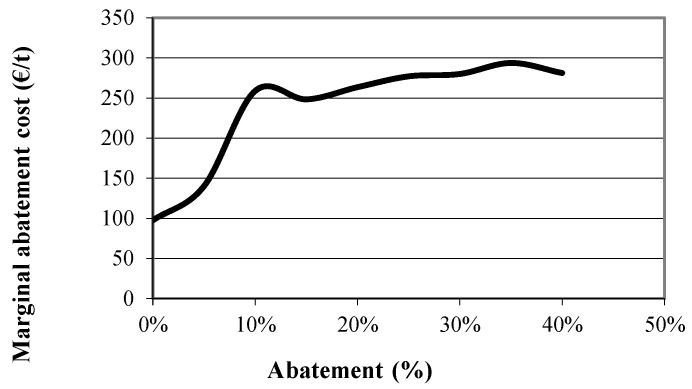
Marginal abatement cost curve of the intensive farm.

Carbon emissions at the optimal farm plan for the extensive, the semi-intensive and the intensive farms were first estimated and are presented in Table 6, Table 7 and Table 8, respectively. The constraint on total emissions was then inserted and the emissions at the new optimal farm plans were again obtained for various levels of abatement (α = 10%, 15% and 20%), through parametric optimization. Emissions per source at various levels of abatement are also presented in Table 6, Table 7 and Table 8. The values of certain variables of the model, that summarize the optimal farm plan at these abatement levels for the extensive, the semi-intensive and the intensive farm, are presented in Table 9, Table 10 and Table 11, respectively. This way, the best abatement strategy for each farm can be identified. Finally, the marginal abatement cost for each of the farms was estimated and the marginal abatement cost curve is built and presented in Figure 3, Figure 4 and Figure 5.

### 3.1. GHG Emissions

As can be seen in Table 6, Table 7 and Table 8, the results of the analysis emphasize the significance of CH_4_ in goat farms. Methane represents 75%, 65% and 52% of total emissions of the extensive, the semi-intensive and the intensive farms, respectively. Methane emissions refer mainly to CH_4_ from enteric fermentation, as the CH_4_ produced from manure management is negligible. Methane is particularly high in the extensive-farming system, where the feeding of livestock is based on grazing. On the other hand in the case of the intensive farm, methane from enteric fermentation is considered low, because of the high amount of compound feed used in the ration. Nitrous oxide emissions from manure management are also a significant source of GHGs in dairy goat farms, since it accounts for 20%, 25% and 34% of total emissions of the extensive, the semi-intensive and the intensive farming systems.

Emissions per kg of goat milk are estimated at 4.08, 2.04 and 1.82 kg of CO2-eq for the extensive, the semi-intensive and the intensive dairy goat production system respectively. The carbon footprint of goat milk is particularly high in the case of the extensive-farming system. On the other hand in the semi-intensive and the intensive farming system emissions per kg of milk are low and comparable to the emissions estimated for cow’s milk (see also [6,41,42]). The reasons for this variation of carbon footprint among the alternative farming systems are the high productivity of more intensive farms and the significant amount of compounds in the ration used in the semi-intensive and the intensive farms, compared to the low productivity of the extensive farms and the grazing/forage-based nutrition.

As can be seen in Table 6, Table 7 and Table 8, when emissions are restricted to various levels, the emissions per kg of produced milk were also reduced, in all farm types. In other words, lower levels of total farm emissions correspond not only to lower milk production levels but also to lower carbon footprint of milk. Specifically, emissions from enteric fermentation per kg of milk are reduced across various levels of abatement, as the result of adopting appropriate feeding practices. Carbon dioxide emissions from purchased feed are also reduced, which indicates that either farms purchase fewer feedstuffs or purchase feedstuffs that cause fewer emissions when they are produced. These results depict the optimal abatement plan for goat farms, as will be discussed in more detail in the next paragraph.

### 3.2. Abatement Cost and Strategies

Table 9, Table 10 and Table 11 summarize the optimal farm plan of the extensive, semi-intensive and intensive farm. The tables emphasize the fact that in all cases abatement has a negative impact on farm gross margin, particularly in the case of the intensive farm. Specifically, in the intensive farm 10% and 20% reduction in emissions result in 9% and 18% loss in farm gross margin, respectively. The reduction in farm gross margin in the case of the semi-intensive and the extensive farm, when emissions are reduced by 10% and 20%, is about 2% and 5%, respectively.

The impact of abatement in the case of the intensive farm can be explained by the high productivity and specialization of the farm in milk production. Over 85% of the gross production value of the farm comes from milk production, while milk yield/goat and price of milk/kg are very high (520 kg/goat and 0.73 €/kg of milk, respectively). The high productivity of the intensive farm is also emphasized by the high gross margin per goat in Table 11.

Furthermore, the analysis indicates that, in all farm types, the mitigation of GHGs is primarily achieved by the reduction of the herd size, especially when high levels of abatement are imposed. Specifically, in the extensive farm 10% and 20% abatement leads to 7% and 16% reduction in livestock size, respectively. In the case of the semi-intensive farm the reduction in livestock size is 8% and 17%, while in the intensive farm the reduction is even higher, 9% and 19%, respectively. These findings are in accordance with previous studies regarding mitigation of GHGs in livestock farms (see for example [17,23]).

However, adjustments in farming practices may also achieve some level of abatement. Specifically, as previously commented, the results indicate that the reduction of purchased feed and their substitution with on-produced feed is a strategy that can lead to lower emissions in all goat production systems. However, as can be observed in the case of the extensive farm, the use of purchased cottonseed cake is suggested as good practice to reduce emissions, since the amount consumed per goat either remains stable or increases across the various levels of abatement. The explanation for this finding lies in the fact that the inclusion of oil-rich feedstuffs in the ration of ruminants can lead to lower CH_4_ emissions from enteric fermentation [43].

Furthermore, in the case of the semi-intensive and the extensive farms, the use of pastureland/shrubland and grassland is also included in the optimal farm plans, when abatement is imposed. In these low productivity farms, the use of pasture and grassland and the switch to on-produced feed reduce the feeding cost and compensate at a great extent the loss in total gross margin caused by abatement.

These results are also confirmed by the marginal abatement cost curve of the extensive, the semi-intensive and the intensive farm type which are presented in Figure 3, Figure 4 and Figure 5, respectively. As can be seen in the figures the marginal abatement costs of the extensive and the semi-intensive farms are very low compared to the intensive farm. Specifically, the marginal abatement cost of the extensive farm is 11 €/t at the 95% level of the original emissions, 35 €/t at 80% and 76 €/t at 60%. In the case of the semi-intensive farm the marginal abatement cost is about 50 €/t, until 30% of the original emissions are abated and reaches 220 €/t at 40% abatement level. On the other hand, in the case of the intensive farm the marginal abatement cost reaches 250 €/t at only 10% abatement, indicating that intensive farms, already achieve the production of low carbon footprint milk and further abatement comes at a higher cost.

Two scenarios are investigated in this analysis, regarding the potential to restore the gross margin of the goat farms that is reduced as the result of GHG abatement. First, the impact of milk price increase is investigated using parametric optimization. The results indicate that a small price increase of about 5%–6% allows the extensive and the semi-intensive farms to maintain their original gross margin and still abate 20% of their emissions. This price increase may for example come as the result of the labeling of milk as a low-carbon product. In the case of the intensive farm the price increase should be 14% in order for the farm to achieve its original gross margin level.

Alternatively, as far as policy measures are concerned, the loss in farm gross margin can be restored if farms are offered compensation/subsidy per productive goat. This compensation should be less than 6 €/goat in the cases of the extensive and the semi-intensive farming system but should reach 56 €/goat in the case of the intensive system, at 20% abatement level. It should be noted, however, that the majority of goat farms in Greece are extensive and semi-intensive farming systems, while only a few farms are characterized as intensive. Therefore, the cost of this policy measure may not be prohibitive, though this should be further investigated.

Finally, it should be mentioned that abatement has a significant impact on labor in all three production systems (see Table 9, Table 10 and Table 11). Specifically, 10% and 20% abatement results in 7%–9% and 16%–19% reduction in required labor inputs of the farms, respectively. This is an important finding, given the fact that the activity is mainly located in less favored areas of the country, where alternative occupations are scarce.

## 4. Discussion of Results and Conclusions

In this study a mixed-integer programming model was used to estimate GHG emissions in dairy goat farms in Greece and explore their abatement opportunities and cost. The analysis is undertaken in three goat farms that represent the extensive, semi-intensive and intensive production systems and takes into account all potential emission sources within the farm as well as pre-chain emissions.

The results of the analysis indicate that in all production systems, the main source of GHG emissions is enteric fermentation. Emissions per kg of milk are particularly high in the extensive farm, mainly because of its low productivity. The analysis also emphasizes that the intensive-farming system can produce milk with very low carbon footprint, while the carbon footprint of milk produced in semi-intensive farms is also relatively low.

Moreover, the analysis also suggests that imposing high levels of abatement unavoidably leads to the reduction of livestock size and, therefore, milk production. However, lower levels of abatement can be achieved by adjusting farming and especially feeding practices. These mitigation practices include the use of oil-rich feedstuffs, like cottonseed cake, in the ration of livestock and the substitution of purchased feed with on produced feed. These findings are important for farmers who are encouraged to adopt not only economically but also environmentally sound farming practices. The substitution of purchased feed with homegrown feed reduces emissions that are associated with their transportation to the farm, while at the same time reduces the feeding cost of farmers. However, such an adjustment in the feed would entail serious adjustments to the production system of farms, including new investments in land and machinery, as well as a different labor usage. Thus, further investigation is required concerning these implications.

As far as the marginal abatement cost is concerned, it is increasing across various levels of abatement and is significantly higher in the case of the intensive farm. The results reveal that the high productivity of the intensive farm causes a significant loss of gross margin when abatement is imposed. The abatement cost of the extensive farm is smaller, because of its smaller milk yield and, therefore, its smaller gross margin per goat. Abatement also results in a significant reduction of labor required in all farm types, which should also be taken into account when designing environmental policy measures.

Moreover, the results of the analysis indicate that the loss in gross margin caused by abatement may be restored by a small milk price increase in the case of extensive and semi-intensive farms. Further investigation is required to establish whether this price increase is possible from the promotion of milk labeled as a low-carbon footprint product. From the policy makers’ point of view, a small compensation offered to farmers per productive goat can also restore the original gross margin of extensive and semi-intensive farms, when abatement is imposed. Finally, it should be emphasized that even though the carbon footprint of milk is higher in extensive farms, other environmental benefits may emerge from these production systems that are beyond the scope of this study but have to be considered when estimating their overall sustainability.

## Figures and Tables

**Table 1:** Livestock feed requirements.

Animal Characteristics	Dry Matter (kg/day)	Digestible Nitrogen (g/day)	Net Energy for Lactation (MJ/day)
Productive goats			
Preservation			
Live weight (kilos)			
50	1.6	40	5.2
60	1.8	46	5.8
70	2.0	52	6.6
Pregnancy			
Live weight (kilos)			
50	1.4	105	8.4
60	1.5	120	9.0
70	1.6	140	9.8
Lactation (per kilo of milk)			
Fat content			
3.0%	-	50	2.8
3.5%	-	55	3.0
4.0%	-	60	3.2
4.5%	-	65	3.4
Male goats			
Live weight (kilos)			
80	2.1	63	8.1
100	2.2	75	9.6
Growing animals			
Age (in months)			
0–1		80	3.2
1–2	0.3–0.6	80	3.6
2–3	0.6–0.8	77	4.2
3–4	0.8–1.0	74	4.6
4–5	1.0–1.1	68	4.9
5–6	1.1–1.2	62	5.1
6–7	1.2–1.3	60	5.2

Source: Zervas et al., 2000 [24].

**Table 2:** Nutritional value of feed.

Type of Feed	Dry Matter (g/kg)	Digestible Nitrogen (g/kg)	Net energy for Lactation (Mj/kg)	Fiber Matter (g/kg)
Maize for grain	0.880	0.073	8.40	0.022
Barley for grain	0.860	0.077	7.60	0.044
Cotton seed	0.922	0.195	7.99	0.211
Alfalfa hay	0.850	0.105	4.10	0.280
Maize silage	0.300	0.018	2.15	0.053
Herbaceous material (pastures)	0.202	0.019	1.13	0.038
Shrubs	0.472	0.021	1.64	0.280
Oat for grazing (grassland)	0.275	0.015	1.45	0.090

**Table 3:** Emission sources considered in the analysis.

Emission Sources	Included in the Analysis	Not Included in the Analysis
Livestock emissions		
Enteric CH_4_	X	
CH_4_ from manure deposited onto pasture	Χ	
CH_4_ from manure management	Χ	
Direct Ν_2_Ο emissions from manure deposited onto pasture	Χ	
Indirect Ν_2_Ο emissions from manure deposited onto pasture	Χ	
Ν_2_Ο emissions from leaching and run-off from manure deposited onto pasture		Χ
Direct Ν_2_Ο emissions from manure management	Χ	
Indirect Ν_2_Ο emissions from manure management	Χ	
Ν_2_Ο emissions from leaching and run-off from manure management		Χ
Crops		
Direct Ν_2_Ο emissions from use of fertilizers	Χ	
Indirect Ν_2_Ο emissions from use of fertilizers	Χ	
Ν_2_Ο emissions from leaching and run-off		Χ
CO_2_ pre-chain emissions associated with the use manufacture and transport of inputs (fertilizers and pesticides)	Χ	
CO_2_ from energy use within the farm	Χ	
Purchased feed		
Direct Ν_2_Ο emissions from use of fertilizers	Χ	
Indirect Ν_2_Ο emissions from use of fertilizers	Χ	
Ν_2_Ο emissions from leaching and run-off		Χ
CO_2_ pre-chain emissions associated with the use manufacture and transport of inputs (fertilizers and pesticides)	Χ	
CO_2_ from energy use required for the cultivation and transport of purchased feed	Χ	

**Table 4:** Main Characteristics of the production systems identified in Greece and of the representative farms used in the analysis.

Characteristics	Farming Systems [2]			Representative Farms		
	Extensive	Semi-Intensive	Intensive	Extensive	Semi-Intensive	Intensive
Farm size	No significant diversification (extensive usually larger)			350 productive goats	300	300
Breeds	Hardy local breeds,	Improved local breeds	Highly productive breeds foreign breeds or local improved breeds	Local breeds	Improved local breeds	Highly productive local improved breeds
Use of pastures-shrublands	About 80% of feeding requirements, supplementary feeding during winter	50% of the feeding requirements	0% of the feeding requirements	75% of the feeding requirements	30% of the feeding requirements	0% of the feeding requirements
Use of concentrates	About 15% of nutritional requirements	Higher than extensive, lower than intensive	Mainly used to satisfy livestock feeding requirements	20% of the feeding requirements	60% of the feeding requirements	62% of the feeding requirements
Annual milk yield (kg/goat)	Estimated for Makedonitiki breed by Kitsopanides [2] at 134	Estimated for Skopelou breed by Kitsopanides [2] at 292	Estimated for Saanen and Alpine breeds by Kitsopanides [2] at 580–625	115	300	520
Level of mechanization (level of usage of equipments (e.g., for preparation of feed, milking machines etc.)	Low	Moderate (usually no milking machine)	Very high	Low (no milking machine)	Moderate (no milking machine)	Very high
Invested capital/goat	Low-low productivity livestock	Moderate	Very high	Low	Moderate	Very high
Prolificacy index (number of kids per goat per birth)	Estimated for Makedonitiki breed by Kitsopanides [2] at 1.14	Estimated for Skopelou breed by Kitsopanides [2] at 1.37	Estimated for Saanen and Alpine breeds by Kitsopanides [2] at 1.72–1.74	1.2	1.5	1.80
Percent of milk income to total farm income	Estimated for Makedonitiki breed by Kitsopanides [2] at 57%	Estimated for Skopelou breed by Kitsopanides [2] at 74%	Estimated for Saanen and Alpine breeds by Kitsopanides [2] at 80%	60%	75%	86%

**Table 5:** Main parameters used in the linear programming (LP) model.

Model Parameter	Extensive Farm	Semi-Intensive Farm	Intensive Farm
Variable costs of cultivated crops (€/hectare)			
Maize for grain	-	2142	1651
Maize for forage	-	-	734
Alfalfa for hay	-	-	1148
Barley for grain	591	-	1071
Crop yield (tones/hectare)			
Maize for grain	-	15	11
Maize for forage	-	-	54
Alfalfa for hay	-	-	15
Barley for grain	3	-	3
Price of purchased feedstuff (€/kg)			
Maize for grain	0.20	0.20	-
Barley for grain	-	0.30	-
Alfalfa hay	0.22	0.22	0.16
Mixture	-	0.40	0.40
Cotton seed	0.25	-	-
Variable cost for livestock (except for feeding and labor) (€/adult goat)	18.96	26,49	39.2
Replacement rate	22%	7%	16%
Average price of meat sold (€/kg)	4.25	2.9	3.43
Average price of milk sold (€/kilo)	0.63	0.60	0.73

**Table 6:** Annual greenhouse gas (GHG) emissions of the extensive farm (in kg of CO_2_-eq).

Abatement (α)	0%		10%		15%		20%	
	Total	Per kg of Milk *	Total	Per kg of Milk	Total	Per kg of Milk	Total	Per kg of Milk
Total GHGs	305,576	4.08	275,025	3.95	259,740	3.92	244,461	3.91
CH_4_ enteric fermentation	226,471	3.03	203,644	2.92	192,858	2.91	181,971	2.91
CH_4_ manure	3207	0.04	2997	0.04	2848	0.04	2690	0.04
N_2_O manure	59,771	0.80	55,901	0.80	53,103	0.80	50,158	0.80
N_2_O fertilizer	899	0.01	450	0.01	450	0.01	450	0.01
N_2_O fertilizer-purchased feed	3250	0.04	2914	0.04	2452	0.04	2050	0.03
CO_2_ energy-purchased feed	8435	0.11	7187	0.11	6371	0.10	5420	0.09
CO_2_ energy-farm	3543	0.04	1536	0.02	1536	0.02	1536	0.02

* These GHGs refer only to milk production. Meat production related GHGs are not presented separately since milk is the main product of the farms. For the allocation of the GHGs between milk and meat the share in the production value is used.

**Table 7:** Annual GHG emissions of the semi-intensive farm (in kg of CO_2_-eq).

Abatement (α)	0%		10%		15%		20%	
	Total	Per kg of Milk	Total	Per kg of Milk	Total	Per kg of Milk	Total	Per kg of Milk
Total GHGs	284,120	2.04	255,708	2.00	238,268	1.98	227,296	1.96
CH_4_ enteric fermentation	182,281	1.31	163,790	1.28	154,348	1.27	146,580	1.27
CH_4_ manure	2767	0.02	2547	0.02	2379	0.02	2302	0.02
N_2_O manure	71,219	0.51	65,691	0.51	61,153	0.51	59,284	0.51
N_2_O fertilizer	1349	0.01	1349	0.01	1349	0.01	1349	0.01
N_2_O fertilizer-purchased feed	6178	0.04	4700	0.04	3961	0.03	3400	0.03
CO_2_ energy-purchased feed	14,662	0.11	11,967	0.09	9413	0.08	8716	0.08
CO_2_ energy-farm	5665	0.04	5665	0.04	5665	0.05	5665	0.05

**Table 8:** Annual GHG emissions of the intensive farm (in kg of CO_2_-eq).

Abatement (α)	0%		10%		15%		20%	
	Total	Per kg of Milk	Total	Per kg of Milk	Total	Per kg of Milk	Total	Per kg of Milk
Total GHGs	332,797	1.82	299,518	1.81	282,878	1.80	266,238	1.79
CH_4_ enteric fermentation	169,926	0.93	152,886	0.92	144,684	0.92	136,352	0.92
CH_4_ manure	3151	0.02	2846	0.02	2700	0.02	2,549	0.02
N_2_O manure	111,501	0.61	100,615	0.61	95,638	0.61	90,150	0.61
N_2_O fertilizer	3609	0.02	3100	0.02	2637	0.02	2372	0.02
N_2_O fertilizer-purchased feed	7183	0.04	6206	0.04	5587	0.04	5075	0.03
CO_2_ energy-purchased feed	23,363	0.13	20,036	0.12	17,882	0.11	16,124	0.11
CO_2_ energy-farm	14,065	0.08	13,829	0.08	13,750	0.09	13,616	0.09

**Table 9:** Optimal farm plan of the extensive farm.

Abatement (α)	0%		10%		15%		20%	
	Total	Per Female Goat	Total	Per Female Goat	Total	Per Female Goat	Total	Per Female Goat
Gross margin (€)	27,271	70	26,738	73	26,389	76	25,957	79
Total labour (hours)	5018	13	4672	13	4441	13	4197	13
Female productive goats	390	1	364	1	346	1	327	1
Purchased cottonseed cake (kg)	4312	11	4265	12	3746	11	4530	14
Barley for consumption (hectares)	1.5	0.00	0	0.00	0	0.00	0	0.00
Purchased barley (kg)	0	0.00	0	0.00	0	0.00	0	0.00
Grassland (hectares)	2	0.01	2	0.01	2	0.01	2	0.06
Purchased alfalfa (kg)	9748	25	7477	21	7586	22	5225	16
Purchased maize (kg)	45,849	118	41,271	113	34,082	99	27,740	85
Fresh grass/shrub (kg)	994,584	2550	902,372	2479	871,492	2519	838,790	2565
Winter pasture (hectares)	50	0.13	50	0.14	50	0.15	50	0.15
Summer pasture (hectares)	100	0.26	100	0.28	100	0.29	100	0.31
Crop cultivation for sale (hectares)	0.5		0		0		0	

**Table 10:** Optimal farm plan of the semi-intensive farm.

Abatement (α)	0%		10%		15%		20%	
	Total	Per Female Goat	Total	Per Female Goat	Total	Per Female Goat	Total	Per female Goat
Gross margin (€)	47,275	136	46,440	146	46,000	151	45,296	157
Total labour (hours)	7140	21	6559	21	6270	21	5,946	21
Female productive goats	348	1	319	1	305	1	289	1
Maize for consumption (hectares)	2	0.006	2	0.006	2	0.007	2	0.007
Pasture (hectares)	50	0.144	50	0.157	50	0.164	50	0.173
Purchased alfalfa (kg)	17,781	51	12,930	41	10,935	36	8,699	30
Purchased maize (kg)	59,692	172	32,812	103	26,419	87	20,570	71
Purchased barley (kg)	0	0	11,334	36	10,010	33	8779	30
Purchased mixture (kg)	33,764	97	27,404	86	24,693	81	22,606	78
Fresh grass/shrub (kg)	294,134	845	293,482	920	294,134	964	291,166	1007
Crop cultivation for sale (hectares)	0		0		0		0	

**Table 11:** Optimal farm plan of the intensive farm.

Abatement (α)	0%		10%		15%		20%	
	Total	Per Female Goat	Total	Per Female Goat	Total	Per Female Goat	Total	Per Female Goat
Gross margin (€)	78.870	259	72,169	261	68,194	261	64,929	263
Total labour (hours)	4071	13	3702	13	3524	14	3345	14
Female productive goats	305	1	276	1	261	1	247	1
Maize for consumption (hectares)	5.5	0.02	4.8	0.02	4.1	0.02	3.7	0.02
Alfalfa for consumption (hectares)	0.8	0.00	1.7	0.01	2.4	0.01	2.8	0.01
Maize silage for consumption (hectares)	0.1	0.00	0	0.00	0.0	0.00	0.0	0.00
Purchased alfalfa (kg)	183,609	602	154,085	558	133,911	513	118,012	478
Purchased mixture (kg)	44,546	146	40,215	146	38,040	146	35,929	145
Barley for consumption (hectares)	0	0.00	0.4	0.00	2.1	0.01	2.6	0.01
Crop cultivation for sale (hectares)	0		0		0		0	
